# Sex differences in cognition, anxiety-phenotype and therapeutic effect of metformin in the aged apoE-TR mice

**DOI:** 10.1186/s13293-025-00684-w

**Published:** 2025-01-20

**Authors:** Yingbin Lin, Xinqun Luo, Fangyu Wang, Huange Cai, Yuanxiang Lin, Dezhi Kang, Wenhua Fang

**Affiliations:** 1 Department of Neurosurgery, Neurosurgery Research Institute, Institute of Neurology, Fujian Provincial Institutes of Brain Disorders and Brain Sciences, The First Affiliated Hospital, Binhai Branch of National Regional Medical Center, Fujian Medical University, Fuzhou, Fujian 350005 China; 2 Department of Ultrasound, The First Affiliated Hospital, Fujian Medical University, Fuzhou, Fujian 350005 China

**Keywords:** Apolipoprotein E4, Metformin, Sex difference, Cognition, Anxiety, Depression

## Abstract

**Background:**

Apolipoprotein E4 (ApoE4) is associated with an increased risk of Alzheimer’s disease (AD), depression, and anxiety, which were reported to improve after the administration of metformin. However, sex influence on the effect of ApoE4 and metformin on cognition and mental health is poorly understood.

**Methods:**

ApoE3-TR and apoE4-TR mice of both sexes were randomly assigned to the normal saline and metformin groups from 13 months to 18 months of age. Behavior tests (MWM, EPM, OFT, TST, FST) were conducted to assess cognition, anxiety, and depression-like behaviors. The mice’s blood glucose was also recorded.

**Results:**

Male aged apoE4-TR mice are more vulnerable to cognitive decline than females. Metformin improves the spatial memory of female, but not male apoE3-TR mice and female apoE4-TR mice while aggravating the cognitive impairment of male apoE4-TR mice. The anxiety-like phenotypes in male apoE4-TR mice are more severe than in male apoE3-TR mice, while metformin ameliorates the anxiety-like behaviors in the male apoE4-TR mice but not in male apoE3-TR mice. In addition, metformin alleviates depression-like behaviors in male and female apoE4-TR mice. The hypoglycemic effect of metformin is insignificant in both male and female apoE4-TR mice.

**Conclusions:**

Male sex exacerbates *APOE4*-related cognitive impairment and anxiety in aged mice and is insensitive to the cognition improvement effect of metformin in the aged apoE3 mice. Male sex with *APOE4* may experience more severe cognitive impairment after treatment with metformin while sensitive to the anti-anxiety effects of metformin. These findings identify sex-specific effects on ApoE4-based dementia, anxiety prevention, and therapy, emphasizing the importance of further sex dimension analyses in vivo and clinical studies.

## Plain language summary

The apolipoprotein E4 (*APOE4*) gene increases the risk of Alzheimer’s disease, depression, and anxiety. The present study examined how ApoE4 and a hypoglycemic drug, metformin, affected male and female mice’s cognition and mental health. The mice were carriers of the *APOE*3 gene (which is less risky) or the *APOE4* gene. They were given normal saline or metformin from 13 to 18 months of age. We found that: (1) Male apoE4 mice showed more cognitive decline than female apoE4 mice as they aged. (2) Metformin improved the spatial cognition of female apoE3 mice but not male apoE3 mice or female apoE4 mice. Metformin worsened the cognitive problems of male apoE4 mice. (3) Male apoE4 mice showed more anxiety-like behavior than male apoE3 mice. However, metformin reduced the anxiety in male apoE4 mice but not in male apoE3 mice. (4) Metformin reduced depression-like phenomenon in both male and female apoE4 mice. (5) ApoE4 reduces the ability of metformin to lower blood sugar in mice, regardless of gender. These findings highlight the importance of considering sex differences in studies of ApoE4-related dementia, anxiety, and treatment.

## Highlights

ApoE4 could aggravate memory decline and anxiety-like phenotype in male aged mice but not females.

Metformin was observed to have a sex-dependent improvement in the spatial memory of aged apoE3-TR mice.

Metformin leads to lower cognitive performance in male apoE4-TR mice and has anti-anxiety effects on them.

ApoE4 blunts metformin’s hypoglycemic impact in aged male and female mice.

## Background

Apolipoprotein E4 (*APOE4*) is a risk factor for developing Alzheimer’s disease (AD) [1]. Late-life depression (LLD) [2], anxiety [3, 4], and cerebrovascular disease [5], are prevalent in the aged population. Metformin, a prescription antidiabetic drug, exerts beneficial effects on cognition, depression, and anxiety in patients and mice [6–9]. The therapeutic effect of metformin on *APOE4*-related cognitive and affective disorders needs further study.

There is a female preponderance in the prevalence of dementia, anxiety, and depression disorders [10–13], emphasizing the importance of sex factors. Moreover, clinical reports show marked inter-sex differences in the relationship between *APOE4* and episodic memory [14], outcomes following several nervous system diseases, such as stroke and traumatic brain injury [15, 16]. In addition, several studies have identified sex-dependent effects of metformin on various health conditions, ranging from cognition [17] to cancer-specific mortality to neuropathic pain [18], in clinical and preclinical settings. Overall, it is crucial to identify sex differences in the effects of *APOE* genotype and metformin on cognitive function and affective disorders.

Previously, we reported that chronic metformin treatment could alleviate apoE4-mediated depression in male mice and improve the cognitive state of the female apoE3-TR mice, but not in their apoE4-TR counterparts [2, 19]. In this study, we focused on the sex differences in cognitive, depressive, and anxiety- phenotypes and effects of metformin therapy in the aged apoE-TR mice. Normal saline or metformin was administrated chronically to the aged apoE-TR mice of both sexes. The cognitive performance of mice was evaluated using the Morris water maze test (MWM). Elevated plus maze (EPM) and open field test (OFT) were used to detect the anxiety-like phenotype, and the depression-like phenotype was tested using the tail suspension test (TST) and forced swimming test (FST). The effects of ApoE4, sex, and metformin on glycemia in aged mice were also investigated. The results could offer an understanding of how ApoE4 and metformin influence cognitive function, depression, and glycemia in the context of both genders.

## Methods

### Animals

Human *APOE* target replacement (TR) homozygous mice, maintained on a C57BL/6J background, were obtained from the Taconic Biosciences (www.taconic.com), in which the endogenous murine *Apoe* gene was replaced with human *APOE3* or *APOE4*. We identified the *APOE* genotypes as previously described [20]. Animal studies were conducted in accordance with the rules and regulations by the Animal Care and Use Committee at Fujian Medical University.

### Drug treatments

Thirteen-month-old male and female apoE3-TR and apoE4-TR mice were randomized into the metformin (Met)/ Normal Saline (NS) group. Metformin (Cat. D150959; Sigma, USA) was dissolved in NS. Mice were administered metformin at a dosage of 300 mg/kg daily through oral gavage for 5 months. Following this treatment period, blood glucose levels were measured, and behavioral tests were conducted.

### Behavior tests

The mice were acclimatized for 30 min before being tested in a dimly lit experimental room.

### Morris water maze test (MWM)

The Morris water maze (MWM) test was used to evaluate the spatial learning and memory of mice as described in a previous protocol [21] with some modifications [22]. Briefly, each mouse received four 1-minute training sessions per day in a circular pool (1.2 m in diameter and 0.5 m in height) from four distinct directions in a random sequence for five days. A hidden round platform was placed in a designated location. On the 6th day, we conducted the memory test with the platform removed and each mouse was allowed one minute to explore the pool.

### Elevated plus maze (EPM)

The elevated plus maze (EPM) test serves as a prominent tool for evaluating anxiety-related behaviors and the efficacy of pharmacological agents in reducing anxiety. The apparatus for the EPM consisted of two opposing open arms, two opposing enclosed arms, and a central elevated platform 50 cm in height. The mice were placed at the central platform and allowed ten minutes to explore the maze. The duration and entries in the open arms were measured.

### Open field test (OFT)

The Open field test (OFT) is a commonly recognized method for assessing anxiety levels, spontaneous activity, and exploratory behavior in animal subjects. In a rectangular chamber (50 × 50 × 50 cm), mice were placed in the center and given 10 min to explore. We measured the time and distance in the central area and the total distances traveled by suspending a video camera over the box.

### Tail suspension test (TST)

The tail suspension test (TST) is widely used to assess depression-like behaviors of rodents. The mouse tail was suspended using adhesive tape for 6 min, and the duration of immobility was measured automatically.

### Forced swimming test (FST)

The forced swimming test (FST) is a well-established method for evaluating depression-related behaviors in rodent models. A clear cylinder with a diameter of 20 cm and a height of 40 cm was filled with water and each mouse was placed inside for 6 min. The total immobility time was recorded.

### Blood glucose

Tail blood was used to test blood glucose levels via a Free-Style glucose meter and monitoring strips (Abbott Diabetes Care).

### Statistical analysis

Data are expressed as means ± standard error of mean (SEM), and statistical analyses were conducted using GraphPad Prism 9.0. The Shapiro-Wilk normality test was used to assess data distribution, and the data were mostly normally distributed. Bartlett’s test was applied to determine the homogeneity of variances. Multiple comparisons were analyzed using a three-way analysis of variance (ANOVA) with Tukey’s post-test. *P* < 0.05 was considered statistically significant.

## Results

### ApoE4 impairs the cognitive function of aged mice in a sex-dependent manner, and metformin ameliorates cognitive decline in a sex- and *APOE*-genotype-dependent manner

**Fig. 1:**
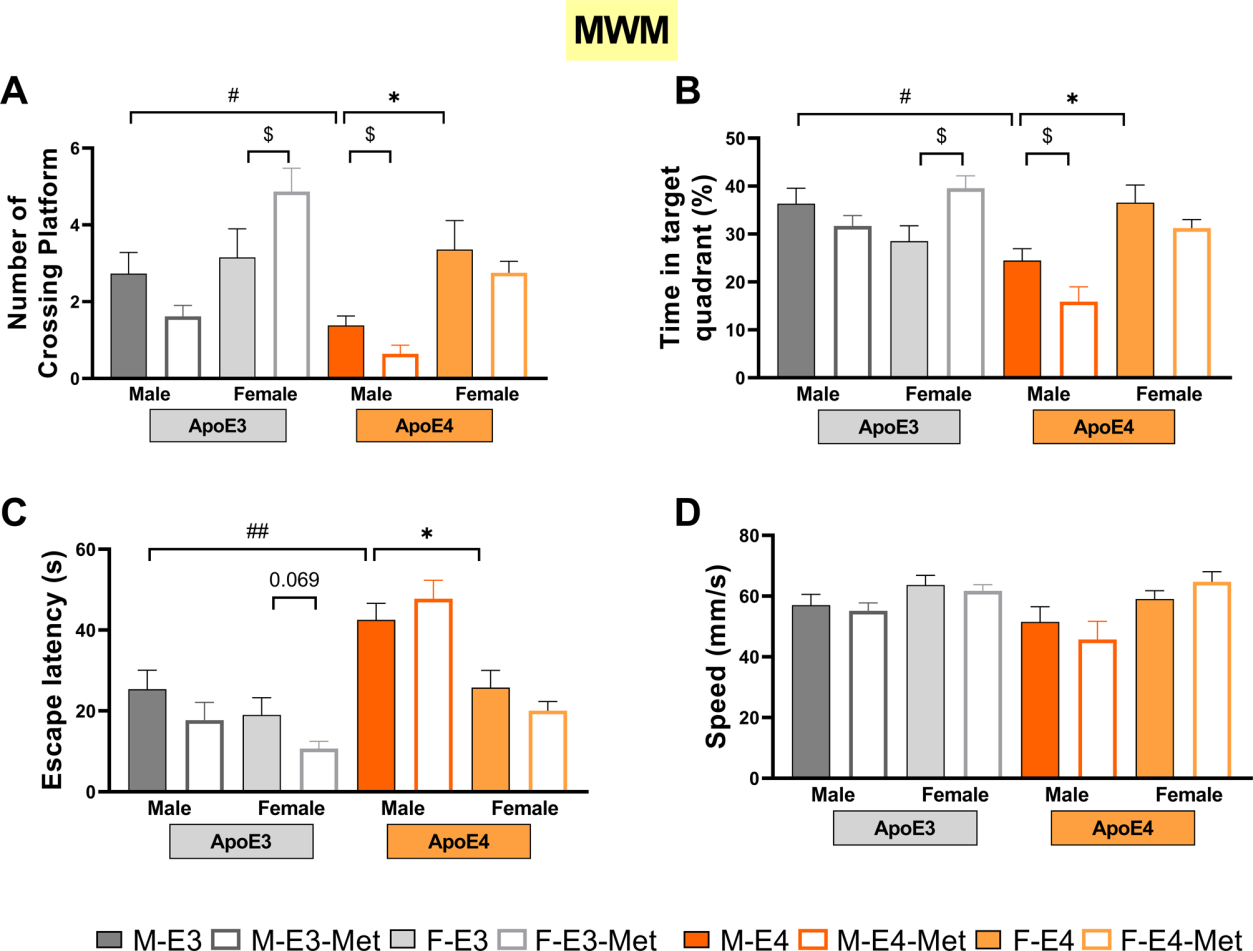
Effects of sex, *APOE* genotype, and metformin on cognitive performances in aged mice. (**A**-**C**) Number of platform-position crossings (**A**), time in the target quadrant (**B**), and escape latency during day 6 (**C**) of the Morris Water Maze (MWM) test of the 18-month-old apoE-TR male and female mice treated with normal saline (NS) and metformin (Met). (**D**) Swimming speed in day 6 of the MWM test. *n* = 12–15/ group. Data are presented as mean ± SEM. Three-way ANOVA was used to calculate statistical significance. * *P* < 0.05 for male vs. female, # *P* < 0.05, ## *P* < 0.01 for apoE3 vs. apoE4, $ *P* < 0.05 for normal saline vs. metformin

Our previous study has shown that no significant differences in spatial memory between apoE3-TR females and apoE4-TR females at 18 months of age, and that metformin improves cognitive performance in the 18-month-old apoE3-TR females but not the apoE4-TR counterparts [19]. To determine the sex-divergent effects of ApoE4 and metformin on cognitive, anxiety-like, and depression-like phenotype in aged mice, male and female apoE3-TR or apoE4-TR mice, were gavaged with metformin from 13 months old to 18months old, and their behavioral manifestation assessed using MWM, EPM, OFT, TST, FST. No significant difference in platform-position crossings, time in the target quadrant, and the escape latency to the platform-position between female apoE3-TR mice and female apoE4-TR mice were observed in MWM (Fig. 1A, B, C). However, male apoE4-TR mice showed fewer platform-position entries, less time spent in the target quadrant, and longer escape latency than male apoE3-TR mice (*P* = 0.042, Fig. 1A; *P* = 0.010, Fig. 1B; *P* = 0.012, Fig. 1C) and female apoE4-TR mice (*P* = 0.023, Fig. 1A; *P* = 0.013, Fig. 1B; *P* = 0.009, Fig. 1C). Moreover, apoE3-TR females treated with metformin crossed the platform location more frequently (*P* = 0.048, Fig. 1A), spent more time in the target quadrant (*P* = 0.012, Fig. 1B), and showed a shorter escape latency (*P* = 0.069, Fig. 1C) than their NS-treated counterparts. However, there was no difference between metformin-treated male apoE3-TR mice and NS-treated males (Fig. 1A-C). Furthermore, compared with the M-E4 mice, the M-E4-Met mice showed fewer target entries (*P* = 0.033, Fig. 1A), less time in the target quadrant (*P* = 0.042, Fig. 1B), and longer escape latency to the platform, though statistically insignificant (*P* = 0.405, Fig. 1C). The groups exhibited no notable differences in swimming speed (Fig. 1D). These data demonstrate that apoE4-TR males are more susceptible to cognitive dysfunction than female ones. Otherwise, metformin improves memory function in the aged apoE3-TR females but not in apoE3-TR males and apoE4-TR females, while metformin seems to aggravate the cognitive decline of male apoE4-TR mice.

### ApoE4 exacerbates the anxiety-like behaviors in aged male mice, which would be alleviated by metformin

**Fig. 2:**
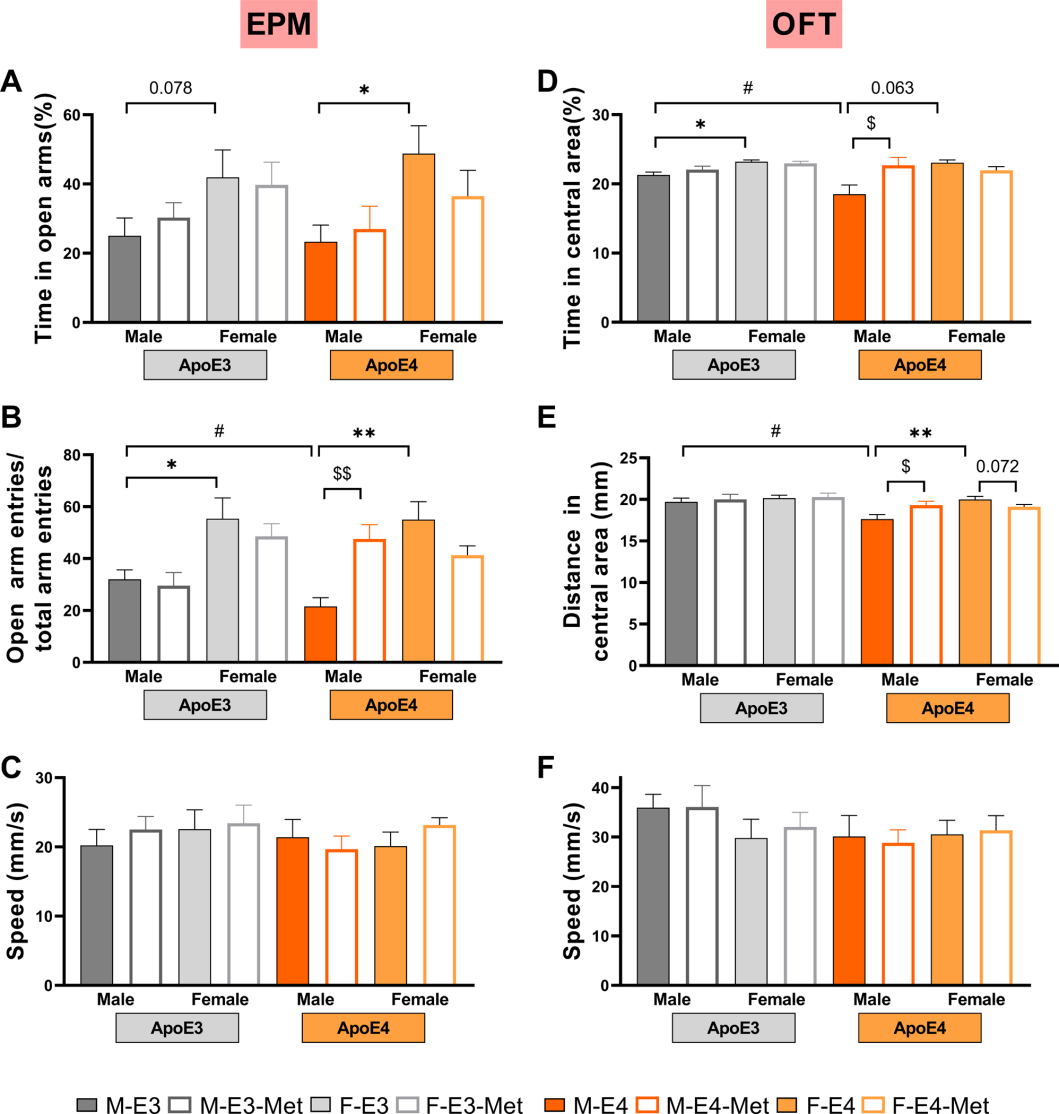
Effects of sex, *APOE* genotype, and metformin on anxiety-like states in aged mice. (**A**, **B**) The time spent in the open arms (**A**) and open arms entries (**B**) for the elevated plus maze (EPM) of the 18-month-old male and female apoE-TR mice treated with normal saline (NS) and metformin (Met). (**C**) Speed for the EPM. (**D**, **E**) The time spent (**D**) and distance traveled (**E**) in the central area for the open field test (OFT) of the 18-month-old male and female apoE-TR mice treated with normal saline (NS) and metformin (Met). (**F**) Speed for the OFT. *n* = 12–15/ group. Data are presented as mean ± SEM. Three-way ANOVA was used to calculate statistical significance. * *P* < 0.05, ** *P* < 0.01 for male vs. female, # *P* < 0.05 for apoE3 vs. apoE4, $ *P* < 0.05, $$ *P* < 0.01 for normal saline vs. metformin

The anxiety-like behavior of mice was tested using EPM and OFT. In EPM, apoE-TR males spent less time in the open arms (M-E3 vs. F-E3, *P* = 0.078; M-E4 vs. F-E4, *P* = 0.013, Fig. 2A) and displayed fewer open-arm entries (M-E3 vs. F-E3, *P* = 0.010; M-E4 vs. F-E4, *P* = 0.002, Fig. 2B) than their *APOE-*genotype-matched female counterparts. Furthermore, apoE4-TR males entered the open arms less frequently than apoE3-TR males (*P* = 0.045, Fig. 2B), which changed with metformin supplementation (*P* = 0.005, Fig. 2B). However, there were no differences in open arms entries frequency and time in the open arms between M-E3 and M-E3-Met (Fig. 2A, B). Similarly, male apoE-TR mice spent less time and displayed less distance in the central area of OFT than their female *APOE-*genotype-matched counterparts (M-E3 vs. F-E3, *P* = 0.035; M-E4 vs. F-E4, *P* = 0.063, Fig. 2D; M-E3 vs. F-E3, *P* = 0.451; M-E4 vs. F-E4, *P* = 0.001, Fig. 2E). This was more pronounced in apoE4-TR groups (Fig. 2D, E) and was rescued by metformin administration (*P* = 0.024, Fig. 2D; *P* = 0.030, Fig. 2E). No significant difference in the speed of EPM and OFT between the groups was observed (Fig. 2C, F). The above results show that the anxiety-like behaviors were present in both apoE4-TR and apoE3-TR male mice; however, metformin ameliorates the anxiety-like behaviors in the apoE4-TR males but not in the apoE3-TR males.

### Metformin alleviates ApoE4-induced depression-like behaviors, and ApoE4 blunts the hypoglycemic effect of metformin both in aged male and female mice

**Fig. 3:**
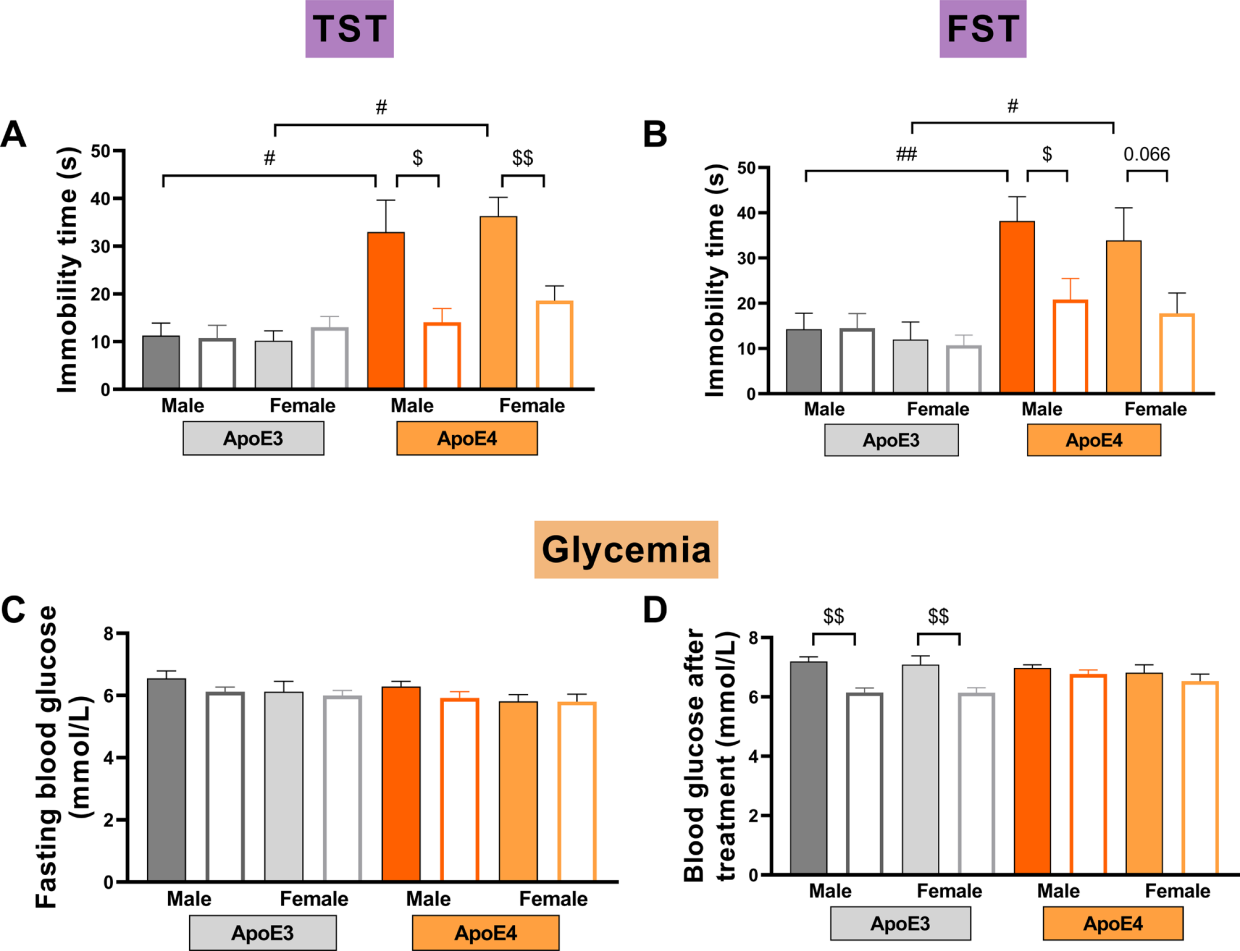
Effects of sex, *APOE* genotype, and metformin on depression-like behaviors and glycemia in aged mice. (**A**) The duration of immobility in tail suspension test (TST). (**B**) The duration of immobility in forced swim test (FST). (**C**-**D**) Blood glucose levels after 10 h of fasting (**C**) and 1 h after administering normal saline or metformin (Met) (**D**) in the male and female 18-month-old apoE-TR mice. *n* = 12–15/ group. Data are expressed as mean ± SEM. Three-way ANOVA was used to assess statistical significance. # *P* < 0.05, ## *P* < 0.01 for apoE3 vs. apoE4, $ *P* < 0.05, $$ *P* < 0.01 for normal saline vs. metformin

The TST and FST were performed to determine the depression-like state of male and female aged mice and evaluate metformin’s potential therapeutic effects. Male and female apoE4-TR mice showed significantly longer immobility time in the TST and FST compared with the sex-matched apoE3-TR mice (*P* < 0.05, Fig. 3A, B), which was significantly alleviated by metformin (*P* < 0.05, Fig. 3A, B). We measured blood glucose after 10 h of fasting and 1 h after administering metformin or normal saline. No significant difference in the blood glucose level at fasting and 1 h after administering normal saline between male and female apoE-TR mice was observed (Fig. 3C, D). According to our previous research, the hypoglycemic effect of metformin is observed in male apoE3-TR mice, while it is not effective in their apoE4-TR counterparts [2], which indicates that ApoE4 demonstrates resistance to the hypoglycemic effects of metformin in aged male mice. This study further investigates the potential sex differences of this phenomenon. Similar results were observed in female aged apoE4-TR mice, that is, the administration of metformin did not lead to a reduction in blood glucose levels in apoE4-TR mice, regardless of sex (Fig. 3D), while it significantly induced hypoglycemic effects in both male and female apoE3-TR mice (*P* < 0.01, Fig. 3D). These results show that metformin alleviates depression-like behaviors and has little hypoglycemic effect on both male and female apoE4-TR mice.

**Fig. 4:**
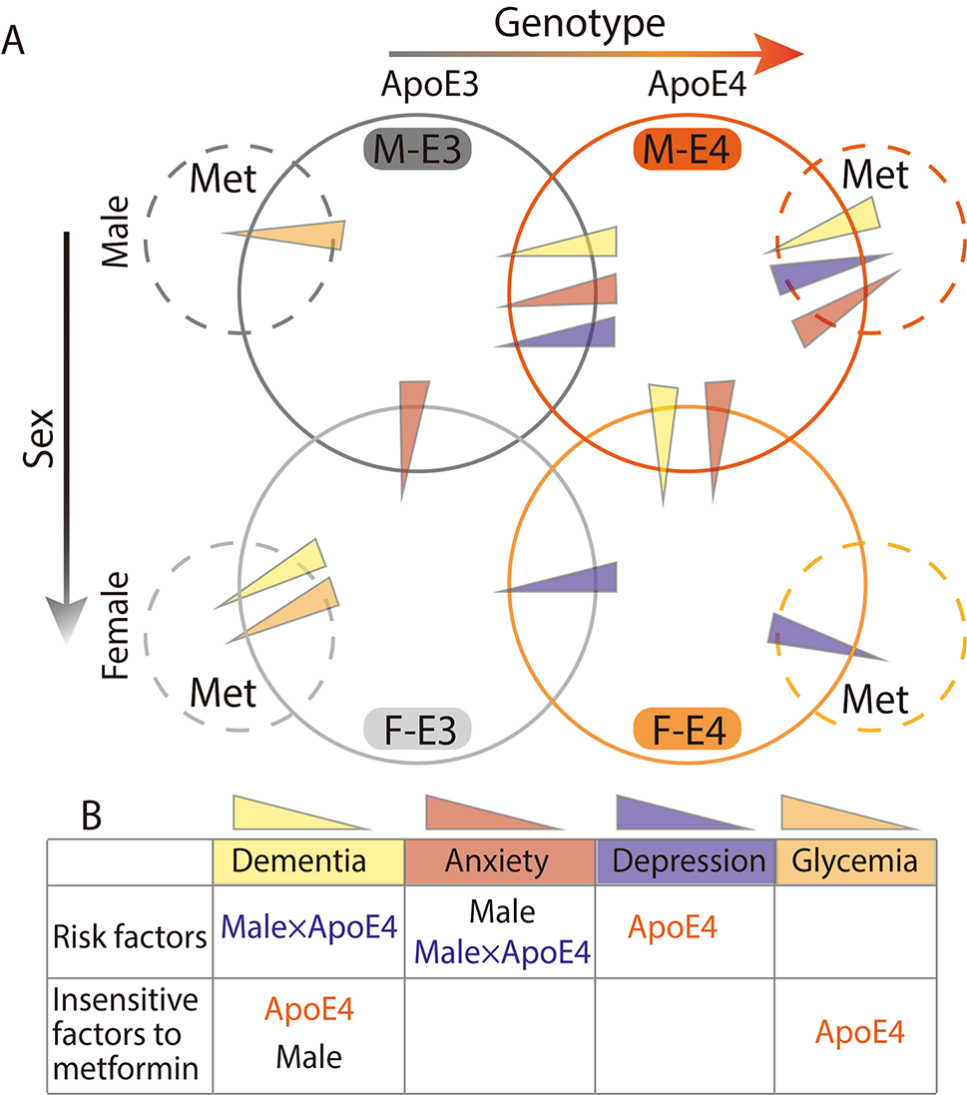
Summary of major findings. (**A**) The dementia, anxiety, depression, and glycemia state of both male and female apoE3-TR and apoE4-TR mice (M-E3, M-E4, F-E3, and F-E4) and their metformin-treated counterparts. The dark gray solid and dashed circles represent the M-E3 and M-E3-Met groups, while the dark orange solid and dashed circles signify the M-E4 and M-E4-Met groups, respectively. Similarly, the light gray solid and dashed circles indicate the F-E3 and F-E3-Met groups and the light orange solid and dashed circles represent the F-E4 and F-E4-Met groups, respectively. The yellow, light red, purple, and orange wedges correspond to dementia, anxiety, depression, and glycemia states, respectively. The height of the wedge indicates the degree of the corresponding state between the two groups in which it is located. (**B**) The risk factors associated with dementia, anxiety, depression, and glycemic states, as well as the factors that reduce susceptibility to metformin treatment for these conditions in aged mice. The term “Male×ApoE4” indicates that the ApoE4 genotype interacts with the male sex, leading to an increased likelihood of developing dementia or anxiety. M, male; F, female; E3, ApoE3; E4, ApoE4; Met, metformin

## Discussion

The study showed sex differences in *APOE4*-related cognitive impairment and anxiety-like phenotype. Male sex is a risk factor for *APOE4*-related cognitive impairment and anxiety in aged mice. Furthermore, metformin improves the memory function of the aged apoE3-TR mice in a sex-dependent manner; that is, the benefits of metformin in memory were only observed in female apoE3-TR but not male mice. More interestingly, metformin lowers cognitive ability in male apoE4-TR mice, while it has anti-anxiety effects on male apoE4-TR mice. No sex differences were found in *APOE4*-related depression and the anti-depressant effect of metformin. There was also no sex difference in the ApoE4 blunted effect on the hypoglycemic effect of metformin. These findings document the sex-specific effect of ApoE4 on cognitive and affective disorders and identify sex- and *APOE*-genotype-divergent effects on the influence of metformin on cognitive performance and anxiety-like behavior.

Our study used a naturally aged apoE-target replacement homozygous mice model to reveal that aged male apoE4-TR mice developed cognitive impairment earlier than female apoE4-TR mice (Fig. 1). The results of a relatively large cohort study involving community-based aged participants show that the *APOE4*-related cognitive impairment effect in men was more severe than that in women, especially in the *APOE4* homozygotes [14], which agrees with our study. It seems that ApoE4 has a dramatic effect on cognition in male sex. However, the results seem to be the opposite when it comes to the cognitive impairment group or Aβ-exposed AD animal models. Wang et al. showed that female carriers of the *APOE4* allele showed significantly steeper episodic memory decline than their male counterparts among the mild cognitive impairment (MCI) individuals, but not the normal cognition or AD group [23]. Furthermore, female ApoE4/3xTg mice experienced earlier and more severe cognitive impairment than male mice [24]. According to these observations, *APOE*4 may exhibit different pathology in different sexes. Several studies found the effect of *APOE4* on cortical thickness [24], cerebral glucose metabolism [25], macrophage inflammatory responses [26], and β-site APP cleavage enzyme (BACE1) expression [24] were influenced by sex, which would provide mechanistic insight into the ApoE4×sex interaction. These studies highlight the complex interplay between ApoE4 and sex on cognitive function, suggesting that further researches are needed to understand their interactions fully.

In addition to the vulnerably of the male sex to ApoE4-mediated dementia, male aged mice carrying *APOE3*, which represent the wildtype mice, are insensitive to the cognitive improvement of metformin (Fig. 1), which were observed in female aged mice carrying *APOE3* in our previous work [19] and in aged wildtype mice [27]. This shows the sex-dependent antidementia effects of metformin in aged mice. Clinical and animal studies have shown that males are less likely to benefit from metformin on cognitive function [28, 29]. In diabetics receiving metformin for more than six months, metformin was also associated with deterioration of cognition [30]. According to Chaudhari and his colleagues, metformin treatment resulted in cognitive impairment in young and aged male C57BL/6 mice. These learning and memory impairments were nearly exclusive to male mice [17]. More importantly, chronic metformin treatment significantly induced memory decline in male mice carrying *APOE4* in the current study (Fig. 1), suggesting that males, especially *APOE4* carriers, should monitor cognitive function during metformin therapy. In addition, clinical studies show that men with *APOE4* alleles have poorer outcomes after ischemic stroke and traumatic brain injury [15, 16]. The totality of the above evidence suggests that when males were present with ApoE4, they were not only prone to cognitive impairment but also more to adverse drug reactions and poor disease outcomes, which may be partly due to the interaction between ApoE4 and testosterone.

Women reported higher prevalence rates of anxiety disorders than men [31, 32]. Rodent studies are inconsistent regarding stable sex differences in anxiety-like behavioral tests due to variables like species, strain, age, and testing conditions [10]. We found that 18-month-old apoE-TR males experienced more severe anxiety-like behaviors than their female counterparts. Specifically, males spent a smaller percentage of time on the open arms of EPM (Fig. 2A) and in the central area of OFT (Fig. 2D) and had a lower open arm entry in the EPM (Fig. 2B) and less distance traveled in the central area of OFT (Fig. 2E). Another study focused on the anxiety-like state of apoE-TR at 12 months of age. It documented that male apoE4 mice tended to spend less time in the open arms of EPM and enter the open arms less frequently, but it was not statistically significant [33]. Moreover, our findings support studies showing more severe anxiety-like states in male rats in the OFT [34, 35] and the EPM [34–36]. The anxious behavior of aged male rats could be rescued by castration before puberty [34], suggesting testosterone production in males likely mediates sex differences in anxiety-like behavior in aged rats. Another study reveals a role for oxytocin systems and the corticotropin-releasing hormone (CRH) pathway of the medial prefrontal cortex (mPFC) in regulating male-specific anxiogenic behavior [37]. As the suicide-related disability-adjusted life years attributable to anxiety disorders in males is twice that in females [38], additional studies of the circuit and hormonal mechanism underlaid male-specific anxiety-like behavior will be crucial in the development of sex-specific therapies.

Studies on the correlation between *APOE4* and susceptibility to anxiety have produced varying results. Some studies have observed no differences in anxiety levels between *APOE4* carriers versus noncarriers [39]. A prospective cohort study in older individuals (>60 years) reported that carriage of the *APOE4* allele in men, but not in women, was associated with the elevated anxiety trajectory [40]. Patel et al. reviewed human studies that examined anxiety in the AD population and found that the presence of the *APOE4* allele is related to increased anxiety at all AD stages, particularly at MCI stages [41]. ApoE4 appears to be more likely to cause anxiety in aged patients with cognitive decline and in males. We documented similar phenomena in apoE4-TR mice; the male aged apoE4-TR mice, suffering from cognitive decline, presented with more severe anxiety-like phenomena than apoE3 counterparts, but not females (Fig. 2B, D, E). Diabetes patients treated with metformin had a lower incidence of anxiety disorders [42, 43]. Moreover, the anxiolytic effects of metformin have been observed in mouse models of anxiety associated with various diseases [7, 9]]. Our study showed that the anxiety-like behavior of male aged apoE4-TR mice was improved by metformin. However, the mechanisms underlying apoE4-related anxiety and the antianxiety effect of metformin warrant further study.

Two longitudinal studies suggest that cognitively intact *APOE4* carriers are not at heightened risk for developing depression over 7.7 or 12 years among those who were aged 21–86 (mostly 50–69) or 20–64 years at baseline [44, 45]. However, other studies have shown that the frequency of the *APOE4* allele is significantly higher in individuals with late-onset depression (LOD) [46–48] but not in early-onset depression (EOD) [48]. Moreover, a 9-year prospective population-based study by Skoog et al. revealed that the presence of *APOE4* is associated with more severe depressive symptoms, the onset of minor depression, and any form of depression in individuals aged 70 to 92 years (average age of 73.8 years) who initially did not have depression [49]. The reasons for the discrepancy between these studies may be related to study design issues, such as the age range of subjects investigated, sample size, ethnicity, widely varying criteria to determine depression status, and so on. In Skoog et al.’s study, the subjects were considerably older than those in other studies mentioned above [44, 45]. Similarly, our previous works indicate that apoE4-TR mice aged 3, 8, and 12 months do not display depression-like behaviors, in contrast to those aged 18 months, which do exhibit such behaviors [2, 50, 51], that is ApoE4 is a risk factor for late-onset depression (LOD). We further found no sex differences in apoE4-mediated LOD and the anti-depression effect of metformin in the present work.

There are several limitations about this study that should be addressed. First, although sex differences in ApoE4-related cognitive decline and anxiety-like behavior are characterized, the circuits and mechanisms that contribute to these differences remain unclear. Second, the research involved the administration of a single dose of metformin, the effects of multiple doses of metformin on the behavior of different *APOE* genotypes and sexes need to be further explored.

In summary, our data show the sex-specific effects of *APOE4* on cognition and anxiety-like behavior and document sex- and *APOE* genotype-dependent metformin effects on memory and anxiety-related state (Fig. 4). This suggests that sex and *APOE* genotype are critical determinants for future cognitive and affective investigations.

## Data Availability

No datasets were generated or analysed during the current study.
